# Evaluation of KRAS inhibitor-directed therapies for pancreatic cancer treatment

**DOI:** 10.3389/fonc.2024.1402128

**Published:** 2024-05-10

**Authors:** Szu-Aun Long, Amber M. Amparo, Grace Goodhart, Syed A. Ahmad, Andrew M. Waters

**Affiliations:** ^1^ Department of Surgery, University of Cincinnati College of Medicine, Cincinnati, OH, United States; ^2^ Department of Cancer Biology, University of Cincinnati, Cincinnati, OH, United States

**Keywords:** PDAC, KRAS inhibitor, pancreatic cancer, KRAS, KRAS inhibition

## Abstract

Despite significant advancements in the treatment of other cancers, pancreatic ductal adenocarcinoma (PDAC) remains one of the world’s deadliest cancers. More than 90% of PDAC patients harbor a Kirsten rat sarcoma (KRAS) gene mutation. Although the clinical potential of anti-KRAS therapies has long been realized, all initial efforts to target KRAS were unsuccessful. However, with the recent development of a new generation of KRAS-targeting drugs, multiple KRAS-targeted treatment options for patients with PDAC have entered clinical trials. In this review, we provide an overview of current standard of care treatment, describe RAS signaling and the relevance of KRAS mutations, and discuss RAS isoform- and mutation-specific differences. We also evaluate the clinical efficacy and safety of mutation-selective and multi-selective inhibitors, in the context of PDAC. We then provide a comparison of clinically relevant KRAS inhibitors to second-line PDAC treatment options. Finally, we discuss putative resistance mechanisms that may limit the clinical effectiveness of KRAS-targeted therapies and provide a brief overview of promising therapeutic approaches in development that are focused on mitigating these resistance mechanisms.

## Introduction

RAS genes (KRAS, HRAS, NRAS) are the most frequently mutated oncogene family, with mutations occurring in 19% of patients with cancer (1). KRAS mutations comprise 77% of the RAS mutations in human cancer, making it the most frequently mutated RAS isoform (1). KRAS mutations are selectively enriched in lung, colon, and pancreas cancers – the three deadliest cancers in the United States (2, 3). Roughly one of three lung cancers, one of two colon cancers, and nine of ten pancreatic cancers have KRAS mutations (1). Since the initial discovery that KRAS is mutated in human cancers (4), there has been intense research efforts to develop targeted anti-KRAS therapies. For decades, efforts to target mutant KRAS, aptly nicknamed “the beating heart of cancer” (5), were unsuccessful (6). Thus, KRAS was deemed “undruggable” (7, 8), and developing drugs to target KRAS became one of the Holy Grails of cancer research (9). However, milestone advancements in the field have led to successful development of direct RAS inhibitors (10), culminating in the FDA granting accelerated approval to the first direct KRAS inhibitors, sotorasib (11) and adagrasib (12).

Pancreatic ductal adenocarcinoma (PDAC) is the most common (90%) and lethal pancreatic malignancy, with a KRAS mutation frequency of 94% and an abysmal five-year survival rate of only 13% (3, 13, 14). Until 2011, the 10-year survival rate for pancreatic cancer patients had remained stagnant at 6% for 40 years (15). Despite an improvement in survival over the past decade, the overall prognosis for PDAC remains grim, with survival averaging less than a year following diagnosis (16, 17). Unfortunately, the incidence of PDAC has been rising over the past decade, and because increases in incidence are outpacing increases in survival, PDAC is projected to be the second leading cause of cancer-specific mortality in the United States by 2030 (18).

The incremental, yet steady increase in five-year survival for patients with pancreatic cancer is mainly due to significant advances in the treatment of resectable and borderline resectable disease (19–22). Improvements in radiologic imaging, advances in surgical techniques, and the use of neoadjuvant chemotherapy in downstaging PDAC to allow for surgical resection are all contributing factors (23). In the last 8 years, the five-year survival rate of locally or regionally staged pancreatic cancer has increased from 38% to 60% while the five-year survival rate of patients with distant disease, the stage at which most patients are diagnosed, has only improved from 2% to 3% (3, 24). Thus, effective treatment for patients with advanced pancreatic cancer remains a crucial unmet clinical need. As mutations in KRAS are not only the major initiating event (25, 26), but are also required for maintenance of established tumors (27, 28), deployment of successful KRAS inhibitors for pancreatic cancer patients holds tremendous therapeutic promise for all pancreatic cancer patients, particularly those with distant disease who are in dire need of new therapeutic approaches. In this review, we offer an overview of current PDAC treatment options, discuss KRAS signaling, review the clinically relevant KRAS inhibitors for PDAC patients, summarize the clinical efficacy and safety profile of various KRAS-directed inhibitors to date, and provide an overview of promising therapeutic approaches aimed at overcoming resistance to KRAS-directed therapy.

## Current treatment strategies for PDAC

The primary treatment options for patients with PDAC are surgery and chemotherapy. At the time of diagnosis, only 15-20% of PDAC patients are eligible for surgical resection (20, 29, 30), which is considered the cornerstone of curative treatment. The majority of PDAC patients are diagnosed at a locally advanced or metastatic stage (3), and patients who present at these stages are typically not surgical candidates. Therefore, treatment options have traditionally been limited to chemotherapy. However, these chemotherapy regimens are often associated with intolerable side effects (31) and poor quality of life (32). Regardless of surgical eligibility, the clinical care of PDAC patients involves systemic chemotherapy. In recent years, two chemotherapy regimens have emerged as first-line options for the treatment of metastatic PDAC – FOLFIRINOX (folinic acid, fluorouracil, irinotecan, and oxaliplatin) (31) and gemcitabine + abraxane (33). Patients who progress on one of these regimens or are unable to tolerate the treatment side effects can be considered for the other for second-line treatment. However, there is no recommended universal second-line regimen, and around half of patients fail to ever receive any additional therapy after progressing on one first-line regimen (34, 35). For those patients that are treated with second-line gemcitabine + abraxane after failing FOLFIRINOX treatment, the overall response rate (ORR) is only 2.9%, with 85% of patients experiencing grade 3-4 treatment-associated adverse events (TRAEs) (36). Comparatively, and as discussed in this review, all KRAS inhibitors that have entered clinical trials for patients with pancreatic cancer have higher ORRs and lower frequencies of TRAEs than second-line chemotherapy treatment, regardless of whether the inhibitors are in a Phase I dose escalation study or FDA-approved (**Table 1**).

**Table 1:** Summary of KRAS inhibitor and chemotherapy trials in pre-treated PDAC patients.

	Drug Regimen	Trial	N	ORR (%)	DCR (%)	OS (months)	PFS (months)	Grade 3-4 TRAEs (%)
KRAS inhibitors	Adagrasib (MRTX849)	KRYSTAL-1 (NCT03785249)	21	33	81	8.0	5.4	27
	Sotorasib (AMG-510)	CodeBreaK (NCT03600883)	38	21	84	6.9	4	16
	RMC-6236	Preliminary (NCT05379985)	46	20	87	NA	NA	11
Chemotherapy combinations	nal-IRI+5-FU/LV	NAPOLI-1 (NCT01494506)	117	17	52	6.1	3.1	NR
	Folinic acid, fluorouracil, oxaliplatin (OFF)	CONKO-003 (NCT00786058)	77	NR	NR	5.9	3	43
	Gemcitabine + abraxane	QUILT-3.010 (NCT01834235)	40	2.9	28	6.6	2.7	85

N, number of patients; ORR, overall response rate; DCR, disease control rate; OS, overall survival; PFS, progression-free survival; TRAEs, treatment-related adverse events.

## Mutant KRAS is required for initiation and maintenance of PDAC

KRAS is the major oncogenic driver for PDAC tumorigenesis and is also required for tumor maintenance. Most commonly, KRAS mutations arise in either the acinar or ductal cells of the pancreas (37). KRAS-mutant acinar cells undergo a differentiation process termed acinar-to-ductal metaplasia (ADM), and then either KRAS-mutant ductal cells or KRAS-mutant acinar cells that have undergone ADM can further transition to microscopic lesions termed pancreatic intraepithelial neoplasms (PanINs) over the course of several years (38). KRAS mutations are found in >90% of low-grade PanINs (25). However, KRAS mutations alone do not frequently lead to PDAC. Notably, in recent autopsy studies of donors (>20 to <80 years old) with no known pancreatic disease, most donors possessed PanIN lesions, and almost all PanINs from otherwise healthy pancreata possess KRAS mutations (39, 40). These data strongly suggest that KRAS mutant-PanINs are more common than initially anticipated, and mutant KRAS alone is not sufficient to drive invasive PDAC. In mice, when Kras mutations are coupled with mutations in Tp53, Cdkn2a, or Smad4, PanINs frequently progress to metastatic PDAC (41–44). In humans, KRAS also cooperates with loss-of-function mutations in TP53, CDKN2A, and SMAD4 to drive PDAC progression. Importantly, these are the only four gene mutations that occur with frequencies >15% in PDAC (13).

Mutant KRAS not only plays a critical role in the initiation of PDAC development, but mutant KRAS is also required to maintain tumor growth (25–28). Several studies have shown that in mouse models, ablation of mutant KRAS in established tumors promotes PDAC regression (27, 28, 45), emphasizing the promising therapeutic potential for KRAS-directed inhibitors.

## KRAS signaling

KRAS is a lipidated, small guanosine-5’-triphosphate hydrolase (GTPase) molecular switch that regulates cell proliferation, differentiation, and survival from the inner leaflet of the plasma membrane (46). In healthy cells, KRAS cycles between an active guanosine triphosphate (GTP)-bound (ON) state and an inactive guanosine diphosphate (GDP)-bound (OFF) state. In quiescent cells, KRAS is predominantly GDP-bound. When activated by growth factors, receptor tyrosine kinases (RTKs) stimulate guanine nucleotide exchange factors (GEFs) like SOS1 to exchange GDP for GTP in the active site of KRAS, facilitating a conformational change in plasma membrane-associated KRAS that promotes transient downstream effector interactions. The integrated output of these signaling pathways is pro-proliferative. KRAS, as a small GTPase, has a very slow intrinsic GTP hydrolysis rate that is greatly potentiated by GTPase-activating proteins (GAPs) like neurofibromin 1 (NF1) and p120RASGAP, which facilitate hydrolysis of the gamma phosphate of GTP, resulting in inactive, GDP-bound KRAS (47, 48).

Hotspot gain-of-function KRAS mutations occur at amino acids G12, G13, and Q61, and abrogate the ability of GAPs to assist in hydrolysis of GTP in the active site of KRAS, leading to persistently GTP-bound KRAS and hyperactivation of downstream effector pathways (2, 13). Dysregulation of these signaling pathways can lead to uncontrolled cell growth, and if left unchecked, invasive cancer. While KRAS can signal through many key effector pathways, the RAF-MEK-ERK mitogen-activated protein kinase (MAPK) and the PI3K-Akt-mTOR (PI3K) pathways are two of the best studied due to their role in tumorigenesis (6).

In pancreatic cancer, there are three main reasons to suggest the RAF-MEK-ERK MAPK pathway is the key KRAS downstream effector pathway facilitating tumorigenesis. First, the small fraction of KRAS wild-type (WT) PDAC tumors possess mutually exclusive, activating MAPK mutations 44% of the time, whereas PI3K pathway mutations occur in fewer than 10% of KRAS WT PDAC tumors (49). In contrast, mutations in PI3K often co-occur with KRAS mutations, suggesting PI3K alone is not a strong tumorigenic driver in PDAC (50, 51). Second, analogous to mutant Kras, Braf^V600E^ mutations can initiate PanIN formation in mice, and when coupled with a gain-of-function Tp53^R270H^ mutation, drive invasive PDAC. In contrast, Pik3ca^H1047R^ activating mutations did not lead to obvious PanIN formation (25, 52). Finally, KRAS^G12R^ mutations are the third most common KRAS mutation and selectively enriched in PDAC, despite their inability to directly engage PI3Kα, further supporting the notion that KRAS-dependent PI3K signaling is not critical for PDAC (13, 53).

## All RAS isoforms are not equivalent

For decades, pervasive dogma in the field was that all mutations in all three cancer-relevant RAS isoforms (KRAS, HRAS, and NRAS) were functionally equivalent. There were several reasons for this line of reasoning. First, hotspot mutations in all three RAS genes occur at G12, G13, and Q61. Second, there is 80-90% amino acid sequence identity and tremendous structural similarity between the G-domain of the RAS proteins. Third, all RAS proteins need to be post-translationally prenylated for plasma membrane localization. Fourth, mutant RAS proteins persistently activate the same key downstream effector pathways. Fifth, all mutant RAS genes frequently observed in patients similarly transform NIH3T3 cells (2, 13, 54).. This notion of RAS isoform equality persisted, in part, because of the lack of well-validated tools and reagents for RAS research (55, 56). With the advent of better reagents and tools, the field is now acknowledging that each RAS isoform has unique properties (56). However, the “all RAS isoforms are equivalent” ideology ultimately led to the initiation of three farnesyltransferase inhibitor clinical trials for pancreatic cancer patients that were unsuccessful (57–59).

The farnesyltransferase inhibitor trials were initiated based, in part, on HRAS-mutant mammary and salivary carcinoma mouse models (60), where it was determined that if HRAS is not farnesylated, it cannot be properly localized to the plasma membrane and therefore cannot signal. The field now acknowledges that these clinical trials were fundamentally flawed, because KRAS (and NRAS), unlike HRAS, can be alternatively prenylated by geranylgeranyl-transferase to localize to the plasma membrane (61). Another distinction between the RAS isoforms is that despite the similarity in amino acid sequence, the RAS genes have tremendous variability in their codon usage, and this distinction has been evolutionarily conserved in KRAS for hundreds of millions of years (62, 63). A prevailing theory is that these nucleotide differences confer disparate translation rates of the RAS proteins and add an important layer of differential RAS protein regulation (62). It has also been reported that KRAS preferentially activates the MAPK pathway whereas HRAS preferentially activates the PI3K pathway (64). These observations, coupled with the observation that KRAS is the RAS gene primarily mutated in the three leading causes of cancer death in the US, have helped home the focus on KRAS (65–67).

## All KRAS mutations are not equivalent

Although it was also long thought that all mutations within a particular RAS isoform were equivalent, it is now known that all KRAS mutations are not equal. Strikingly, although almost all PDAC patients have KRAS mutations, different KRAS mutations in PDAC occur with variable frequencies, and different point mutations can confer a different prognosis. The most prevalent KRAS mutations are G12D (39-41%), G12V (28-34%), G12R (14-16%), Q61H (4-6%), and G12C (1%) (13, 68–70). It is established that patients with KRAS-mutant PDAC have a worse prognosis compared to patients with WT KRAS tumors (26, 71–73). Notably, patients with KRAS^G12D^ tumors have been shown to have worse overall survival compared to patients with KRAS^G12R^-mutant PDAC (74–78). While some studies have found that KRAS^Q61^ mutations were associated with improved survival compared to codon 12 mutations (50, 79), a recent high-powered study analyzing patient data from multiple databases reported KRAS^Q61^- and KRAS^G12D^-mutant PDAC patients have the worst prognosis (78).

**Figure 1:**
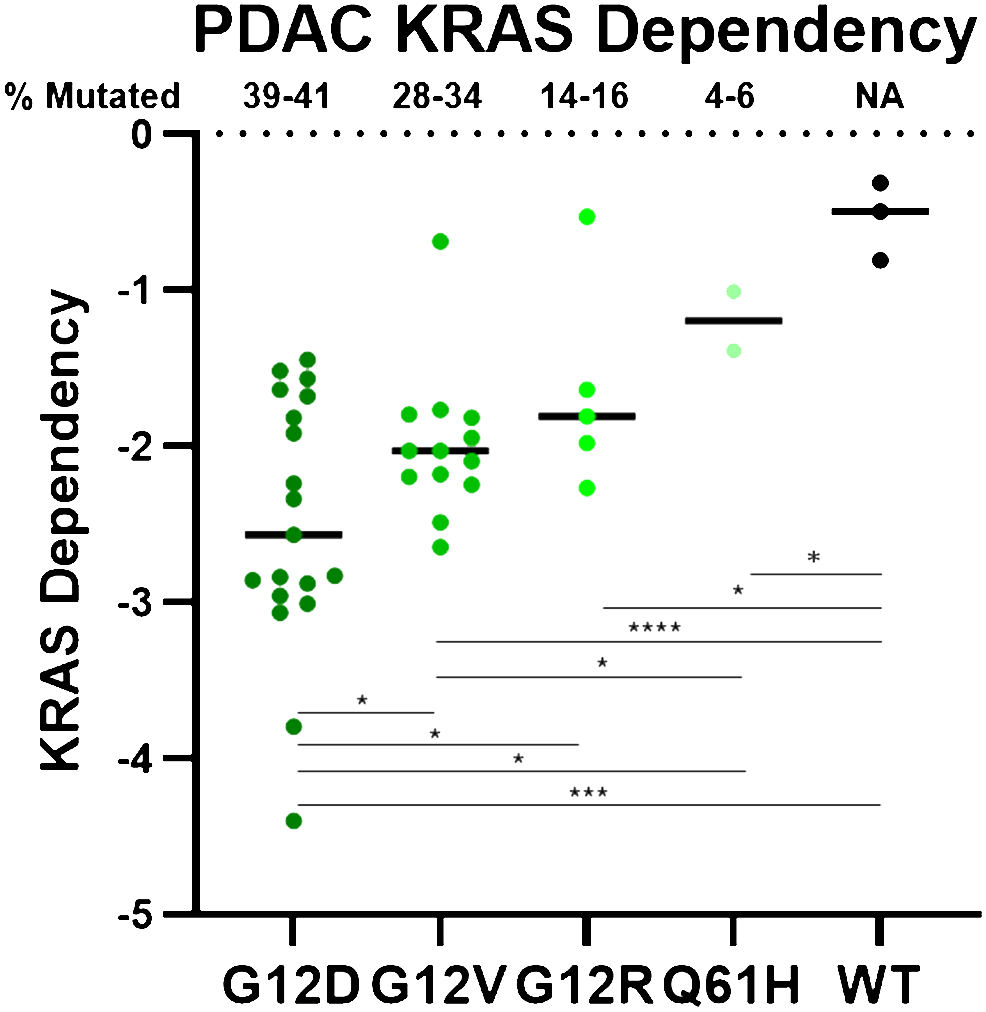
PDAC cell line DepMap data was plotted based on KRAS dependency and stratified by KRAS mutation. Negative scores indicate greater KRAS dependence. Each circle represents one PDAC cell line. **** = p > 0.0001, *** = p > 0.001, * = p > 0.05.

Distinct KRAS mutations also possess distinct biochemical properties that result in important biological differences. Different KRAS mutant proteins vary in their intrinsic and GAP-stimulated hydrolysis rates, as well as their ability to interact with upstream and downstream effectors (47). Notably, although all KRAS mutants are substantially impaired in GAP-mediated hydrolysis, KRAS^G12C^ has intrinsic hydrolysis rates most comparable to KRAS WT, and has been deemed a “fast-cycling” mutant. Similarly, KRAS^G12D^ also maintains some intrinsic hydrolysis activity (47). Further, KRAS^Q61H^ and KRAS^G12R^ are impaired in SOS1 binding (53, 80), and KRAS^G12R^ is impaired in PI3Kα binding (53). These findings, along with other biochemical distinctions, confer unique KRAS mutation-specific vulnerabilities (47, 53, 80–83) that may underlie their unique KRAS dependencies (**Figure 1**) (83–85). Notably, there is a correlation between mutation frequency and KRAS dependency in PDAC cell lines for the most common KRAS mutations (mutation frequencies >1%). While all KRAS-mutant PDAC cell lines are more dependent on KRAS than their WT counterparts, KRAS^G12D^, the most frequently observed KRAS mutation, confers the strongest KRAS dependency, as shown in **Figure 1**. Conversely, KRAS^Q61H^-mutant PDAC cell lines are the least dependent on KRAS. These differential KRAS dependencies may modulate response to targeted, mutation-selective KRAS inhibition.

## Direct inhibitors of KRAS

Developing drugs to directly target KRAS has been challenging. KRAS binds GTP with picomolar affinity, and with millimolar concentrations of intracellular GTP, a reversible GTP-competitive inhibitor is beyond current, and likely future, drug discovery efforts. Secondly, other than the nucleotide binding site, KRAS has a protein structure largely devoid of deep pockets, and is therefore not very amenable to allosteric inhibition (13). These issues, combined with the failures of the farnesyltransferase inhibitors mentioned above (57–59), led to the “undruggable” moniker for KRAS. Thus, many past and current efforts to inhibit mutant KRAS signaling have focused on indirect targeted approaches that have been reviewed elsewhere (6). These approaches have been met with limited clinical success for pancreatic cancer patients. The only targeted therapy approved for PDAC is erlotinib, an epidermal growth factor receptor (EGFR) inhibitor utilized in combination with gemcitabine. The addition of erlotinib to gemcitabine extended survival of PDAC patients by only 12 days (86). Thus, the need for new targeted treatment options is dire.

**Figure 2:**
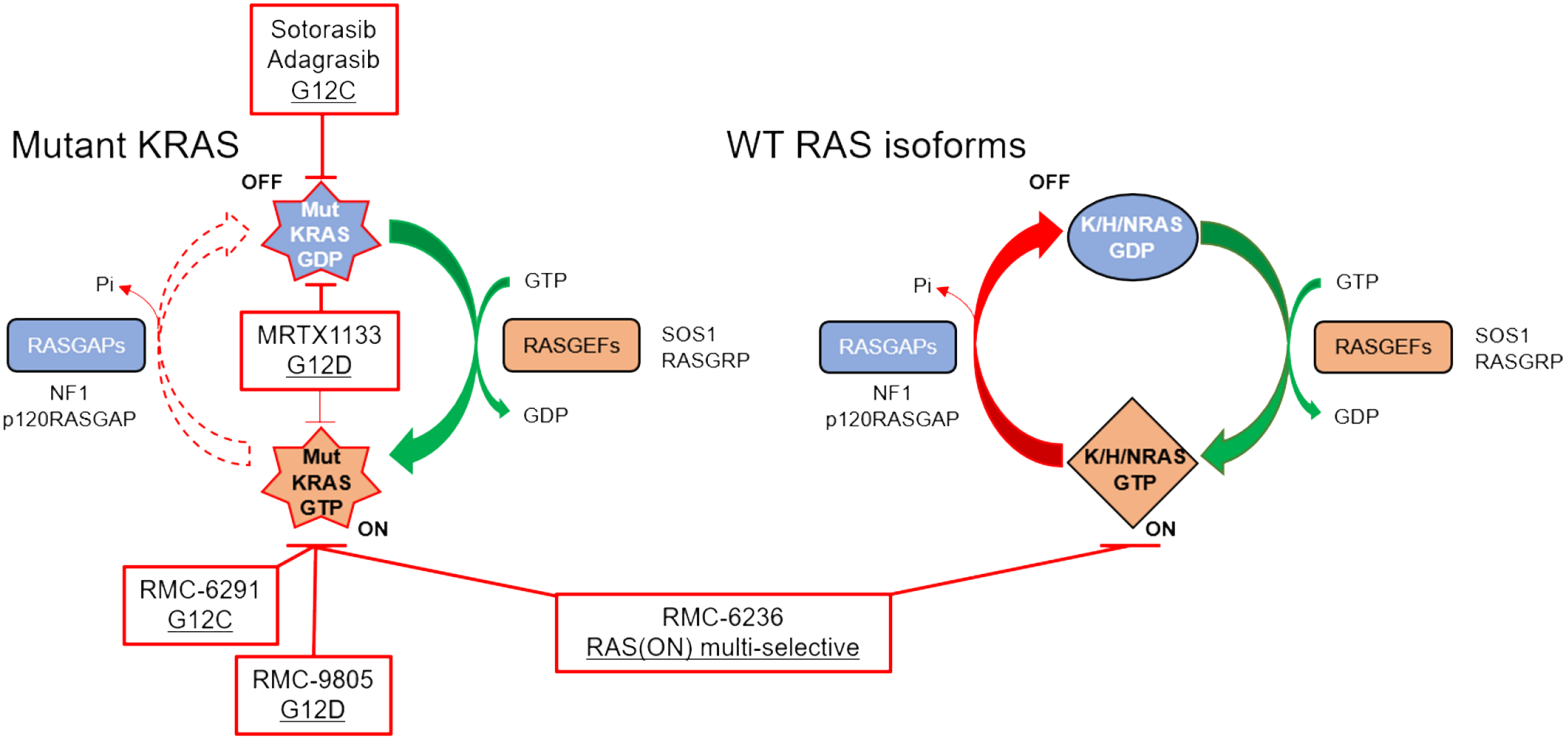
RAS cycling in a KRAS-mutant PDAC cell is driven largely by the mutant KRAS (left), although WT RAS proteins (right) may also be important for intrinsic and acquired resistance. Clinical stage inhibitors discussed in this review are highlighted in red boxes.

The decades of intensity with which drugging KRAS has been pursued, which include unconventional measures such as trying to understand how KRAS folds in microgravity on the International Space Station (87), underscore the potential clinical impact of a successful direct KRAS-targeted therapeutic strategy relevant for pancreatic cancer patients. Remarkably, due to milestone achievements in recent years, the field has now been inundated with KRAS inhibitors, some of which are discussed below. In addition to the two KRAS^G12C^ inhibitors that have been granted accelerated approval, there are at least 17 additional KRAS^G12C^ inhibitors, 5 KRAS^G12D^ inhibitors, and 3 RAS inhibitors targeting multiple mutations undergoing clinical evaluation (88). Many more KRAS inhibitors are in the IND-enabling phase or in preclinical development. While there are many promising KRAS inhibitors in preclinical and clinical development, we focus on reviewing those that we think are most relevant for patients with pancreatic cancer (**Figure 2**, **Table 2**).

**Table 2:** Summary of KRAS inhibitors relevant for PDAC patients.

	Sotorasib	Adagrasib	RMC-6291	MRTX1133	RMC-9805	RMC-6236
Target	G12C			G12D		Multiple
Activation State	OFF	OFF	ON	OFF/ON	ON	ON
Clinical Stage	Approved (NSCLC)		Phase 1* NCT05452717	Phase 1/2 NCT05737706	Phase 1/1b NCT06040541	Phase 1/1b* NCT05379985

*RMC-6291 + RMC-6236 combination trial NCT06128551.

**BI 3706674 is not being evaluated for PDAC patients.

## KRAS^G12C^ inhibitors

In 2013, a landmark paper was published describing a selective, KRAS^G12C^ small molecule that irreversibly and covalently binds KRAS^G12C^ in the GDP-bound state by taking advantage of the nucleophilic and covalent nature of cysteine side chains (10). These small molecules were the predecessors to sotorasib (Amgen), and adagrasib (Mirati Therapeutics), which were the first KRAS inhibitors granted FDA accelerated approval in 2021 and 2022, respectively. Both were approved for KRAS^G12C^-mutant non-small cell lung cancer (NSCLC) (11, 12). This class of compounds is unique in that they covalently modify KRAS^G12C^, binding to GDP-bound KRAS, trapping KRAS in this inactive OFF state. As mentioned above, KRAS^G12C^ is biochemically distinct from other KRAS mutations in that it retains intrinsic GTP hydrolysis rates similar to KRAS WT (47). However, this is a lung-selective approach, as roughly 50% of the KRAS mutations in NSCLC are KRAS^G12C^ (8), whereas only 1% of KRAS mutations in PDAC are KRAS^G12C^.

There was also another breakthrough in the same landmark paper above that has deeper implications for KRAS-mutant PDAC patients (10). The authors determined that the covalent KRAS^G12C^ small molecules sat in a shallow pocket that was not apparent in previous crystal structures of KRAS (10). This pocket has now been further exploited for the development of non-G12C, reversible mutation-selective and pan-KRAS inhibitors that have also entered clinical evaluation, discussed below (89–91).

RMC-6291, under clinical evaluation (NCT05462717) and developed by Revolution Medicines, represents a KRAS^G12C^ inhibitor with a novel mechanism of action. Unlike sotorasib and adagrasib, RMC-6291 and RMC-4998 are tri-complex, covalent RAS(ON) G12C-selective inhibitors that bind to the GTP-bound ON state of KRAS, sterically occluding interactions with downstream effectors (92). This class of inhibitors first binds to cyclophilin A, a chaperone protein, upon entering the cell. On its own, cyclophilin A does not interact with WT or mutant RAS proteins. However, when RMC-6291/RMC-4998 engages cyclophilin A, the binary complex undergoes a conformational change that can then bind to the GTP-bound ON state of KRAS^G12C^, covalently modifying the cysteine and preventing effector signaling. RMC-6291/RMC-4998 does not engage KRAS without first binding to cyclophilin A. Target engagement with RAS(ON) G12C-selective inhibition is more rapid, more potent, and more selective than the FDA-approved KRAS^G12C^ inhibitors (92). The chemical structures of RMC-6291/RMC-4998, and all tri-complex inhibitors derived from this platform, challenge traditional conventions of medicinal chemistry.

## KRAS^G12D^ inhibitors

KRAS^G12D^ is the most common KRAS mutation (39-41%) in PDAC patients, and KRAS^G12D^-mutant PDAC cell lines are significantly more dependent on KRAS for survival than any of the other KRAS-mutant PDAC cell lines (**Figure 1**) (85). This suggests that a KRAS^G12D^ inhibitor for KRAS^G12D^-mutant PDAC patients would have an immense therapeutic impact. Unlike KRAS^G12C^, KRAS^G12D^ lacks the reactive cysteine residue that is easily amenable to covalent modification. However, using the experience gained from developing adagrasib, Mirati Therapeutics performed several structure-based drug optimization studies which led to the discovery of MRTX1133, a reversible KRAS^G12D^ inhibitor currently under clinical evaluation (NCT05737706) (90, 91). MRTX1133 is a potent and selective noncovalent inhibitor that preferentially binds to GDP-bound KRAS^G12D^ at sub-nanomolar concentrations (90). Although MRTX1133 demonstrates preference for inhibiting GDP-bound KRAS^G12D^, it has also been shown to interact directly with GTP-bound KRAS and inhibit the binding of a RAF-RAS binding domain (RBD) peptide to active KRAS^G12D^ (90).

MRTX1133 has shown antitumor efficacy in both *in vitro* and *in vivo* studies. In KRAS^G12D^-mutant PDAC cell lines, MRTX1133 reduced cell viability and inhibited oncogenic KRAS pathway signaling in a dose-dependent manner (90, 91, 93). In tumor xenograft mouse studies with KRAS^G12D^-mutant HPAC cells, intraperitoneal injections of MRTX1133 demonstrated antitumor effects in a dose-dependent manner (90). Near-complete responses (85% tumor regression) were seen in mice who received 30 mg/kg twice daily for 28 days. Additionally, there were no overt signs of toxicity.

Despite aspartic acid being less reactive than cysteine, Revolution Medicines developed RMC-9805 (previously RM-036), a covalent tri-complex RAS(ON) G12D-selective inhibitor. While these data have not yet been peer-reviewed, they were described at the American Association for Cancer Research conference in 2023 (94–96). In preclinical models, RMC-9805 was successful in inhibiting cell proliferation and suppressing RAS pathway activity *in vitro* (94). In mouse KRAS^G12D^ xenograft tumor models, RMC-9805 induced an objective response in 7 out of the 9 PDAC models (95). RMC-9805 is currently being tested in a Phase I trial enrolling patients with advanced KRAS^G12D^-mutant solid tumors (NCT06040541). To date, no peer-reviewed data has been released regarding the efficacy or safety of MRTX1133 or RMC-9805 in PDAC patients.

## RAS inhibitors targeting multiple RAS mutations

Although KRAS^G12D^ is the most common KRAS mutation in PDAC, most PDAC patients have non-KRAS^G12D^ mutations. Thus, an inhibitor targeting multiple KRAS mutations would have the broadest clinical impact for patients with PDAC. Recently, Boehringer Ingelheim developed BI-2865, a noncovalent pan-KRAS inhibitor that targets multiple KRAS mutations (89). As the small molecule can trace its roots to sotorasib, BI-2865 binds to the GDP-bound OFF state of WT KRAS and mutant variants, while sparing the WT NRAS and HRAS isoforms, which is speculated to be important for tolerability in patients. In a panel of 39 cell lines, including 7 WT KRAS cell lines, 24 mutant KRAS cell lines, and 8 WT KRAS cell lines with alterations in upstream signaling, originating from lung, colorectal, or pancreatic cancers, BI-2865 inhibited KRAS activation and downstream signaling in both KRAS WT and mutant models. From these experiments, it was determined that BI-2865 is more potent in KRAS^G12C^-mutant cell lines, followed by KRAS^G12D^-, KRAS^G12V^-, and KRAS^G12R/Q61X^-mutant cell lines. Potency may be partially dictated by KRAS dependency (G12D > G12V > G12R, **Figure 1**). The authors provide some data indicating these preferences may be related to the inactive OFF state-selective drug trapping mechanism of BI-2865 (89), suggesting potencies may be dictated by how rapidly the different mutations enter the GDP-bound state. Consistent with this, the mutation-specific potencies align with intrinsic GTP hydrolysis rates for the different KRAS mutants (G12C > G12D > G12V > G12R > Q61L) (47). By extension, BI-2865 is also highly effective in KRAS WT cell lines (89). BI 3706674, a related compound, is under clinical evaluation for patients with unresectable metastatic KRAS WT-amplified gastric, esophageal, and gastroesophageal-junction adenocarcinoma (NCT06056024).

RMC-7977 and RMC-6236 (Revolution Medicines) are reversible, GTP-binding RAS(ON) multi-selective inhibitors that target all KRAS mutations that arise in patients with PDAC (97, 98). Further, RMC-7977 and RMC-6236 target hotspot NRAS and HRAS mutations, as well as WT KRAS, NRAS, and HRAS. As with RMC-4998/RMC-6291 described above, RMC-7977 and RMC-6236 are tri-complex inhibitors that require cyclophilin A binary complex formation prior to interacting with KRAS, sterically occluding downstream effectors. Targeting all three WT RAS isoforms raised skepticism from experts in the RAS community, due to toxicity concerns based on the perceived necessity for WT RAS signaling in healthy human cells (99). Despite the skepticism among experts for broad toxicity, preliminary data indicate RMC-7977 is well-tolerated with minimal toxicity at doses that generated deep tumor regressions in multiple mouse models (97, 100). Potential reasons for the impressive safety profile may be due to the metronomic nature of RAS or MAPK pathway inhibition in healthy cells as compared to the sustained response in tumor cells (100), the enrichment of cyclophilin A in tumor cells (97), or the ability of RMC-7977 to specifically target the GTP-bound ON state of RAS.

RMC-6236 is currently undergoing clinical evaluation for KRAS^G12A/D/V/R/S^-mutant advanced solid tumor patients, including PDAC patients (NCT05379985) (98). Despite being a Phase I dose-escalation study with a primary goal of identifying a maximum tolerated dose, heavily pre-treated patients appear to be responding well to treatment without overt toxicity, as discussed below. In addition to being utilized as a single-agent treatment, RMC-6236 is also under clinical evaluation as a companion drug to RMC-6291 in combination clinical trials for advanced solid tumor patients harboring KRAS^G12C^ mutations (NCT06128551).

## Efficacy and safety of KRAS inhibitors

Despite the low frequency of KRAS^G12C^ mutations in PDAC, with trial sites across the United States, there were enough PDAC patients enrolled into both the sotorasib and adagrasib trials to evaluate response and toxicity. The efficacy and safety of sotorasib was evaluated in the CodeBreaK studies, which included 38 KRAS^G12C^ pancreatic cancer patients that had failed previous treatments (101). A confirmed partial response was seen in 21% of the patients, with an 84% disease control rate (DCR). The median progression-free survival and overall survival were 4.0 and 6.9 months, respectively. In the KRYSTAL-1 trial, adagrasib was evaluated in 21 patients with unresectable or metastatic PDAC harboring KRAS^G12C^ mutations (102). A partial response was seen in 33% of the patients, with a 100% DCR, and the median progression-free survival and overall survival were 5.4 and 8.0 months, respectively. Preliminary data from PDAC patients with RMC-6236 also look promising based on preliminary, non-peer reviewed data released at ESMO in 2023. Of the clinically evaluable PDAC patients (23 KRAS^G12D^, 11 KRAS^G12R^, 9 KRAS^G12V^, 1 KRAS^G12S^), 20% exhibited a partial response, with an 87% DCR (103). At the time of data release, most patients were still being treated with RMC-6236, with average ongoing treatment duration being 13 weeks (ranging from 5 weeks to 45 weeks). Because most patients are still on treatment, there is currently no data with RMC-6236 on progression-free survival and overall survival. However, a recently published case report describes a Stage IV PDAC patient with liver and peritoneal metastases who was treated with RMC-6236 after failing traditional therapies. Remarkably, after six cycles of RMC-6236 treatment in the dose-escalation study, the patient experienced a confirmed complete response (98).

The direct KRAS inhibitors also appear to be fairly well-tolerated by patients. Overall, 16% of patients treated with sotorasib experienced grade 3-4 TRAEs, with gastrointestinal symptoms and fatigue being the most common (101). Among all patients treated with adagrasib, grade 3-4 TRAEs were observed in 27% of patients and limited to fatigue and QT prolongation (102). Preliminary data from the RMC-6236 single-agent trial reported a favorable response (103, 104). Of the clinically evaluable PDAC patients, only 10% of lung and pancreas cancer patients experienced grade 3-4 TRAEs. This impressive safety profile is surprising given the skepticism in the field regarding the potential toxicity concerns from targeting all of the WT RAS isoforms. These data suggest RAS(ON) multi-selective inhibition with RMC-6236 may be safer than the current KRAS^G12C^ mutation-selective inhibitors that have been granted FDA accelerated approval.

In comparison to the approved regimens of commonly used second-line treatments for advanced PDAC, all KRAS inhibitors that have been deployed for PDAC exhibit greatly improved clinical responses with substantially fewer grade 3-4 TRAEs (**Table 1**) (105–107). In the PRODIGE trial, FOLFININOX was found to have a less favorable side effect profile compared to gemcitabine alone with a higher incidence of grade 3-4 neutropenia, febrile neutropenia, thrombocytopenia, diarrhea, and peripheral neuropathy. Febrile neutropenia led to a treatment-related death, and 46% of patients experienced grade 3-4 neutropenia (31). In the MPACT Phase III trial, 38% of patients treated with gemcitabine + abraxane experienced grade 3 neutropenia, and 17% of patients experienced grade 3 peripheral neuropathy (33). In the NAPOLI-1 Phase III trial, TRAEs led to dose delay, reduction, and/or discontinuation in 73% of the patients who received nal-IRI+5-FU/LV (108). Overall, the safety profiles of direct KRAS inhibitors compare favorably to current second-line treatment options for PDAC.

Although the efficacy and safety of direct KRAS inhibitors must be investigated in larger datasets comparing these novel inhibitors to current standard-of-care treatment paradigms, initial results are highly encouraging. Comparatively, all second-line PDAC chemotherapy treatments have a lower ORR, lower DCR, and higher toxicity profile than all KRAS inhibitor trials for PDAC patients. Thus, it may be warranted to advance KRAS inhibitors to second-line treatment, at minimum, if they are eventually FDA-approved for PDAC patients.

## Resistance mechanisms to KRAS inhibitors

One of the major challenges in treating PDAC is the development of drug resistance, which severely limits the clinical efficacy of chemotherapy regimens (109–111). Intrinsic and acquired resistance will also limit the effectiveness of KRAS inhibitors in patients with PDAC. Invariably, most lung and colon cancer patients treated with KRAS^G12C^ inhibitors relapse due to treatment-induced resistance. Some KRAS^G12C^-mutant patients never respond to KRAS^G12C^ inhibitor treatment due to *de novo* resistance. Unfortunately, due to the low frequency of KRAS^G12C^ mutations in PDAC, there is not enough patient data to understand how pancreatic tumors will adapt to direct targeted KRAS inhibitor therapies. Thus far, identification of putative resistance mechanisms to KRAS inhibition has largely relied on sequencing circulating tumor DNA (ctDNA) from relapsed lung and colon cancer patients, using targeted gene panels (112–116). These putative resistance mechanisms can be characterized into three categories, all of which lead to increased proliferative signaling – 1) upstream signaling events (RTK mutational activation, amplification, and fusions), 2) RAS-level events (KRAS/NRAS mutations or amplifications), and 3) downstream mutations that hyperactivate PI3K and ERK MAPK signaling (mutational loss of PTEN, mutational activation of RAF and MEK, MYC amplification, etc.). Most known resistance pathways converge on a variety of mechanisms that ultimately potentiate or reactivate ERK MAPK signaling.

Currently, the combination of KRAS^G12C^ inhibitors with EGFR inhibitors is being studied in patients with chemotherapy-refractory KRAS^G12C^-mutant metastatic colorectal cancer, and the addition of EGFR inhibitors to KRAS^G12C^ inhibitors has been shown to greatly increase the response rate of KRAS^G12C^ inhibitors (115, 117, 118). Preclinical data also suggests combining MRTX1133 with a pan-ERBB RTK inhibitor may be a useful combination in KRAS^G12D^-mutant PDAC (119). Several preclinical studies have also shown success in overcoming resistance to KRAS^G12C^ blockade by combining KRAS^G12C^ inhibitors with SHP2 inhibitors (120, 121).

In a syngeneic, subcutaneous model of pancreatic cancer, MRTX1133 remodels the tumor microenvironment in mice, shifting the secreted cytokines and chemokines from an immunosuppressive environment enriched for myeloid-derived suppressor cells (MDSCs) to an immunostimulatory environment enriched for CD4- and CD8-positive T-cells (122). Combination treatment with an immune checkpoint inhibitor and RMC-9805 improved the anti-tumor response in the KRAS^G12D^-mutant PDAC model (96). Given that PDAC is an immunologically “cold” tumor, remodeling the tumor microenvironment with KRAS inhibitors has the potential to lead to combinations with immune checkpoint inhibitors.

Putative mechanisms of acquired resistance were only observed in a subset (about half) of the relapsed lung and colon cancer patients, indicating there are many unknown resistance mechanisms yet to be discovered. There are several potential explanations for why many resistance mechanisms remain unknown. First, ctDNA panels are often not comprehensive and often only include a subset of fewer than 100 genes. Second, patients are most often heavily pre-treated before KRAS inhibitor treatment, and their tumors have already adapted to survive in the presence of multiple cytotoxic chemotherapy treatments, confounding the results. Third, upon relapse, many patients are unwilling to agree to additional invasive biopsies, which are more informative than ctDNA. Fourth, non-genetic mechanisms of resistance, like kinome reprogramming and cell-state changes (EMT, adeno-to-squamous carcinoma (114)), are unlikely to be captured with ctDNA. In PDAC, where there is very limited patient data due to the lack of KRAS^G12C^ mutations, adaptation mechanisms to KRAS inhibition are largely unknown.

## Discussion and future directions

The KRAS and pancreatic cancer fields have undergone a paradigm shift in targeting KRAS over the last decade. The field may be nearing a pivotal point with regards to treatment options for pancreatic cancer treatment. Targeted KRAS inhibitors in clinical trials are performing substantially better than current second-line treatment options for pancreatic cancer patients. Thus, if trends hold, and development of KRAS inhibitors continues to improve, targeted KRAS treatments may eventually supplant chemotherapy as the superior line of treatment for patients with KRAS-mutant PDAC.

However, direct KRAS inhibitors rarely lead to complete responses in PDAC patients. Thus, as the new classes of KRAS inhibitors march toward FDA approval, combinations will be required to increase the efficacy of KRAS inhibitors and extend the lives of pancreatic cancer patients. Understanding how pancreatic cancer cells adapt to treatment will be critical, as the field must identify and mechanistically understand both the intrinsic and acquired resistance mechanisms in order to develop successful therapeutic KRAS inhibitor combination approaches.

While there will certainly be many overlapping resistance mechanisms across tissue types, KRAS mutation profiles, and drug classes, there will also likely be distinctions. KRAS^G12C^ is a fast-cycling mutant, with a more rapid intrinsic GTP-hydrolysis rate than other KRAS-mutant proteins (47). KRAS^G12R^ is impaired in binding to PI3Kα, a critical KRAS effector involved in resistance (53). KRAS^Q61H^ does not engage SOS1 (80). KRAS^G12D^-mutant PDAC is significantly more dependent on KRAS than any other KRAS mutation (**Figure 1**). Because of these biochemical and biological differences between the KRAS mutations, some of which may still be poorly understood, it is likely that distinct resistance mechanisms will arise in a KRAS mutation-specific manner. In the era of precision medicine, these patients can now be rapidly identified and stratified for the best treatment options.

There may also be distinct resistance mechanisms to KRAS inhibitors with different mechanisms of action. For example, some of the resistance mechanisms for OFF state inhibitors like adagrasib or sotorasib may be different than those for ON state inhibitors like RMC-6291. For instance, loss of NF1 has been observed in response to OFF state inhibitors, which facilitates more GTP-bound KRAS, preventing inhibitor binding (114). Loss of NF1 may not be a resistance mechanism to ON state inhibitors, as loss of NF1 would instead facilitate more trapping of GTP-bound KRAS. Further, RAS(ON) G12C-selective inhibitors have been shown to overcome resistance mechanisms that have limited KRAS^G12C^ OFF state inhibitors (92, 114). The reverse will also likely be true.

Finally, KRAS-mutant PDAC patients may respond and adapt differently to the mutation-selective inhibitors such as MRTX1133 or sotorasib compared to inhibitors that target multiple mutations like BI-2865 or RMC-6236. Increased activity or mutational activation of RTKs antagonizes mutation-selective inhibitors by reactivating ERK through the WT RAS isoforms (123). Because the WT RAS isoforms are targeted by RAS(ON) multi-selective inhibition, upstream compensatory reactivation of ERK through RTKs may be blunted. In line with this, data indicate compensatory ERK rebound is delayed/abrogated with RMC-7977 as compared to mutant-selective inhibitors (97). Pan-KRAS and RAS(ON) multi-selective inhibitors like BI-2865 and RMC-7977 have also been shown to target many of the second-site RAS mutations that occur as a resistance mechanism to mutation-selective inhibitors (89, 97, 114, 124). Importantly, RAS(ON) multi-selective inhibition has been shown to overcome clinically relevant resistance mechanisms to mutation-selective KRAS inhibitors (97). Evaluating KRAS inhibitor combination approaches and understanding the nuances associated with each KRAS mutation and the related drug mechanisms of action will be the next critical step needed to achieve prolonged tumor responses for patients with KRAS-mutant pancreatic cancer.

## Author contributions

S-AL: Conceptualization, Investigation, Writing – original draft, Writing – review & editing. AA: Investigation, Writing – original draft, Writing – review & editing. GG: Investigation, Writing – original draft, Writing – review & editing. SA: Writing – review & editing. AW: Conceptualization, Data curation, Funding acquisition, Investigation, Resources, Supervision, Writing – original draft, Writing – review & editing.
